# The Phage Mu Middle Promoter P_m_ Contains a Partial UP Element

**DOI:** 10.1534/g3.114.013607

**Published:** 2015-02-02

**Authors:** Ji Ma, Martha M. Howe

**Affiliations:** Department of Microbiology, Immunology & Biochemistry, University of Tennessee Health Science Center, Memphis, Tennessee 38163

**Keywords:** bacteriophage Mu, Mu promoter P_m_, UP element, prokaryotic transcription, regulation of gene expression

## Abstract

There are three phases of transcription during lytic development of bacteriophage Mu: early, middle, and late. Transcription from the middle phase promoter P_m_ requires the activator protein Mor. In the presence of Mor, transcription from P_m_ is carried out by the *Escherichia coli* RNA polymerase holoenzyme containing σ^70^. A Mor dimer binds to two 5-bp inverted repeats within a 16-bp element centered at −43.5 in P_m_, replacing the normal −35 element contacted by RNA polymerase (RNAP). In this study random and targeted mutagenesis of the sequence upstream (−88 to −52) of the Mor binding site was performed to determine whether P_m_ also contains an UP element for binding of the RNAP α subunit, thereby stimulating transcription. The results demonstrated that mutations upstream of −57 had no effect on P_m_ activity *in vivo*, assayed by expression of *lacZ* fused downstream of a wild-type or mutant P_m_. Mutations at positions −57 through −52 led to decreased transcription from P_m_, consistent with the presence of an UP element. In DNase I footprinting and gel mobility shift assays, paired mutations at positions −55 and −54 did not affect Mor binding but decreased the synergistic binding of Mor with histidine tagged α (His-α), indicating that His-α binds to P_m_ in a sequence- and/or structure-specific manner. Taken together, these results demonstrate that P_m_ has a strong proximal UP element subsite, but lacks a distal subsite.

In *Escherichia coli* K-12, promoters that are recognized by RNA polymerase (RNAP) containing the “housekeeping sigma factor” σ^70^ generally consist of a −35 hexamer, −10 hexamer, and a 17+/−1 bp spacer between them (Hawley and McClure 1982). Multiple bases of the consensus sequences for these hexamers are: −35 (TTGACA) and −10 (TATAAT), and increased promoter strength often correlates with a greater number of bases that match these hexamers. There are other less well-defined interactions between RNAP and the spacer bases, and mutations in the spacer can decrease promoter activity at some promoters (Siebenlist *et al.* 1980; Auble *et al.* 1986).

The UP element, a very A/T-rich region located upstream of the −35 hexamer, is bound by the C-terminal domain of the α subunit (αCTD) of RNAP and increases *rrnB* P1 promoter activity up to 30-fold (Ross *et al.* 1993). At least one-third of stable RNA promoters and 4% of mRNA promoters may contain such UP elements (Estrem *et al.* 1999). In addition, the number of promoters with at least partial UP elements is even larger, because promoters with less AT-rich UP element-like sequences still exhibit UP-element function (Ross *et al.* 1998; Estrem *et al.* 1999). There are two subsites within the UP element: promoter proximal and distal; either can function independently, although the proximal subsite may exert a larger effect on promoter activity. The consensus sequence of the full UP element and its subsites (Figure 1) were identified by *in vitro* selection and contain mostly A and T tracts (Estrem *et al.* 1998, 1999). Some promoters contain another element, the extended −10 motif, which has bases TGn located immediately upstream of the −10 hexamer. This sequence is recognized by σ^70^ (Keilty and Rosenberg 1987; Chan and Busby 1989) and drives transcription efficiently without a −35 hexamer or activator proteins.

**Figure 1:**
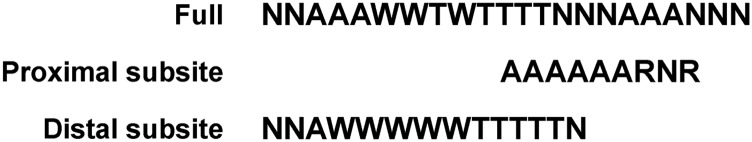
UP element consensus sequences. Consensus sequences of the full UP element and both proximal and distal subsites were identified by in *vitro* selection (Estrem *et al.* 1999). A specific base is indicated when it was present in more than 70% of the sequences; R(A/G) or W(A/T) is used when those two bases were present in more than 95%; N is used when those two circumstances do not apply.

Lytic development of phage Mu occurs with sequential synthesis of proteins: first those involved in replicative transposition, and later morphogenesis, DNA modification, and cell lysis (Marrs and Howe 1990). Phages often orchestrate their development by using phage-encoded activator proteins or sigma factors that are synthesized in and control different stages of gene expression. The Mu activator proteins Mor and C turn on middle and late transcription, respectively (Marrs and Howe 1990; Mathee and Howe 1990. The middle promoter, P_m_, has a recognizable −10 hexamer but lacks a −35 hexamer and requires Mor to initiate middle transcription (Mathee and Howe 1990). Mor consists of 129 amino acid residues (14.7 kDa) and binds as a dimer to a dyad-symmetrical sequence element in P_m_. This 16-bp element contains an inverted repeat of 5 bp each, separated with a G/C-rich hexanucleotide spacer (Artsimovitch and Howe 1996). The αCTD binds to an A/T-rich region just upstream of the Mor binding site, and interactions with Mor increase αCTD binding (Ma and Howe 2004). Two mutations in this region changed promoter activity: −57G to T increased promoter activity twofold and −54A to T decreased promoter activity by half, suggesting that this region might be involved in P_m_ function (Artsimovitch and Howe 1996). The goal of this study was to determine whether the region of P_m_ upstream of the Mor binding site contained an UP element and to identify the role of specific upstream DNA sequences in P_m_ activation. We addressed these goals by making mutations in upstream regions of P_m_ and assaying their effects on promoter activity and binding of Mor and His-α to P_m_ DNA.

## Materials and Methods

### Media, chemicals, and enzymes

Media used included M9 minimal medium with 0.2% casamino acids, Luria broth (LB), and MacConkey lactose plates with only half the normal amount of lactose. Sources for ampicillin (Ap), chloramphenicol (Cm), isopropyl β-D-1-thiogalactopyranoside (IPTG), *o*-nitrophenyl β-galactoside, agarose, and radiolabeled [γ-^32^P]ATP (3000 Ci/mmol) are in the article by Mo and Howe (2014). Sources of enzymes used are in that same paper. Protein gel reagents were from Bio-Rad Laboratories. Oligonucleotides (Table 1) were synthesized by Integrated DNA Technologies, Inc. The N-terminal hexa-histidine tagged α subunit of RNAP(His-α) was overproduced and purified as described previously (Ma and Howe 2004). Purified proteins were generously provided by the following individuals: His_6_-αCTD and untagged αCTD (derived by thrombin cleavage from His_6_-αCTD) from Muthiah Kumaraswami; Mor from Yongkai Mo; and RNAP from Ding Jin (Ma and Howe 2004).

**Table 1:** Primers for P_m_ mutagenesis and sequencing

Primer	Sequence	Coordinates*^a^*	Comments
JM1	GAAATACCGCCAGTACCAGCCCTCACTTCT**N***^b^*TAAA**N**AGTA	top strand	To introduce mutations at -57 and -52
		−87 to –48	
JM2	GAAATACCGCCAGTACCAGCCCTCA**N**TT**N**T**N**TAAACAG	top strand	To introduce mutations at –62, –59 and –57
		−87 to –46	
JM3	GAAATACCGCCAGTACCAGCCCTC**N**C**NN**C**N**GTAAACAG	top strand	To introduce mutations at –63, –61, –60 and –58
		−87 to –46	
JM4	GAAATACCGCCAGTACCA**NNNNNNNN**TTCTG	top strand	To introduce mutations at position –69 to –62
		−87 to –57	
JM5	GAAATACCGCCAGTACCAGCCC**N**C**N**CTTCTG	top strand	To introduce mutations at –65 and –63
		−87 to –57	
JM6	GAAATACCGCCAGTACCAGCCCTCACTTCT**NNNNNN**AGTA	top strand	To introduce mutations at positions –57 to –52
		−87 to 48	
JM7	GTCATAGCTGTTTCCTGTGTGA	bottom strand	Downstream of *Bam*HI, for sequencing P_m_ mutants
JM39	GAAATACCGCCAGTACCAGCCCTCACTTCTAAAAAC	top strand	To introduce mutations –56TA*^c^* (T to A) and –57GA G to A
		−87 to –52	
JM40	GAAATACCGCCAGTACCAGCCCTCACTTCTGT**SS***^d^*ACAGTAA	top strand	To introduce mutations at –55 and –54
		−87 to –47	
JM41	GAAATACCGCCAGTACCAGAAATAAATTCTGTCCACAGT	top strand	To introduce mutations –68CA, –67CA, –66CA, –64CA, –62CA, –55AC and –54AC
		−87 to –49	
MEG4	GGCGAATTC*^e^*ATGCGACGGCTGAAATACCGCCAGT	top strand	for cloning with *Eco*RI site near 5′ end
		−98 to –74	
MEG5	GGCGAATTCATGCGACGGCNNNNNNNNNN*^f^*CCAGT	top strand	AT-rich from –88 to –79 with *Eco*RI site near 5′ end
		−98 to –74	
MEG6	GGCGAATTCATGCGACGGCNNNNNNNNNNCCAGT	top strand	GC-rich from –88 to –79 with *Eco*RI site near 5′ end
		−98 to –74	
MEG7	GGCGAATTCATGCGACGGCTGAAATACCGNNNNNNNNNNCCCTCA	top strand	AT-rich from –78 to –69 with *Eco*RI site near 5′ end
		−98 to –63	
MEG8	GGCGAATTCATGCGACGGCTGAAATACCGNNNNNNNNNNCCCTCA	top strand	GC-rich from –69 to –78 with *Eco*RI site near 5′ end
		−98 to –63	
MLK7	CCTGGATCCGTACGGTTATTCATCACAG	bottom strand	For cloning with *Bam*HI site near 5′ end
		+10 to –9	
IRI21	TGGGGATCGGAATTATCGT	top strand	Upstream of *Eco*RI, for sequencing P_m_ mutants
IRI22	AACTGGCGGCTGTGGGATT	bottom strand	Downstream of *Bam*HI, for sequencing P_m_ mutants

a Coordinates indicate the base locations in the P_m_ sequence relative to the transcription start site as +1.

b N indicates an equal molar mixture of all four bases (A_1_T_1_G_1_C_1_).

c When specific base changes were made, such as –56TA, the first base is the wild-type (T) and the second is the mutant base (A) that was substituted.

d S indicates an equal molar mixture of G+C bases.

e Restriction sites for cloning are underlined.

f N indicates AT-rich primer synthesis corresponding to A_2_T_2_G_1_C_1_ or GC-rich primer synthesis corresponding to A_1_T_1_G_2_C_2_.

### Bacterial strains

Strains used for phenotypic assays of P_m_ activity are derivatives of *E. coli* K-12 strain MH13312 [*mcrA ∆proAB-lac thi gyrA endA hsdR relA supE*44 *recA/F*′ (*pro^+^ lacI*^q1^ *∆lacZY*)] (Artsimovitch and Howe 1996). Strains MH13335, MH13339, and MH15001 are derivatives of MH13312 containing just pKM78 (MH13315) (Artsimovitch and Howe 1996), both pKM78 and pIA12 (MH13339) (Artsimovitch and Howe 1996), and both pKM78 and pMM1 (MH15001) (Mitchell and Howe, unpublished data). Strain RLG3538 [F^−^ *ompT hsdS*_B_ (r_B_^−^m_B_^−^) *gal dcm* λDE3/pLysS/pHTT7f1-NHα] contains plasmid pHTT7f1-NHα encoding His-α in an *E. coli* B strain BL21 background (Gaal *et al.* 1996). Strain MH10713 [*ompT hsdS*_B_ (r_B_^−^ m_B_^−^) *gal dcm* λDE3/pLysS], the Howe lab version of strain BL21 with λDE3 and pLysS (Studier and Moffat 1986), was transformed with plasmid pKM90 (Mathee and Howe 1993) to create strain MH13231 (Ma and Howe 2004) used for overproducing Mor protein. The λDE3 in strains MH10713 and RLG3538 contains the T7 RNA polymerase gene under the control of the IPTG-inducible P*_lac_*_UV5_ (Yanisch-Perron *et al.* 1985; Bachmann 1987).

### Plasmids and plasmid construction

**Figure 2:**
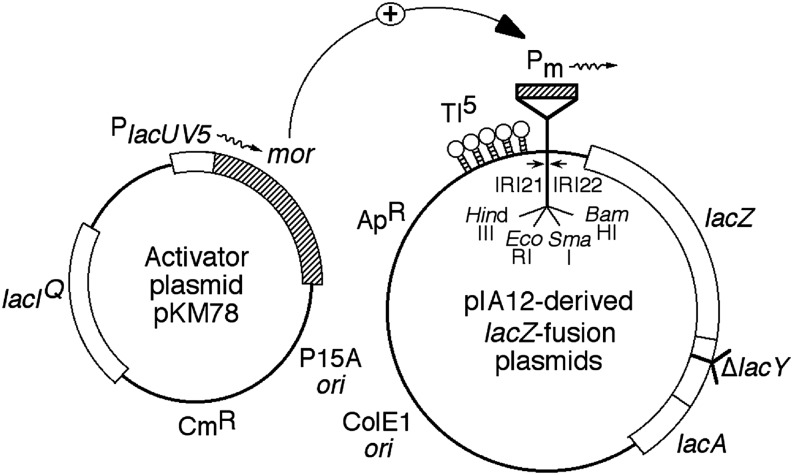
Middle promoter two-plasmid *in vivo* assay system for promoter activity. DNA fragments with promoters, in this case P_m_ sequence −98 to +10, are cloned between the *Eco*RI and *Bam*HI sites in pIA12 to generate the P_m_-*lacZ* fusions. Plasmid pKM78 contains a WT *mor* gene under the control of the P*_lacUV5_* promoter. When cells contain both plasmids, the addition of isopropyl β-D-1-thiogalactopyranoside (IPTG) leads to induction of Mor expression from pKM78. Mor then binds and activates transcription from the WT or mutant P_m_ promoter cloned in pIA12, leading to synthesis of β-galactosidase at levels proportional to P_m_ activity. This figure was published previously by Artsimovitch and Howe (1996).

The promoter cloning plasmid, pIA12 (Figure 2), contains a *Hin*dIII-*Eco*RI-*Bam*HI linker upstream from a promoter-less *lacZ* gene (Chiang and Howe 1993; Artsimovitch and Howe 1996). Plasmid pMM1 contains P_m_ sequence from −98 to +10 cloned into the *Eco*RI and *Bam*HI sites in pIA12 (Chiang *et al.* 1993). Plasmid pKM78 (Figure 2) expresses Mor under the control of P*_lac_*_UV5_ (Mathee and Howe 1990). Plasmid pKM90 contains the *mor* gene downstream of the strong T7 promoter and ribosome binding site in pT7-7 (Tabor and Richardson 1985; Mathee and Howe 1993; Ma and Howe 2004). Plasmid pHTT7f1-NHα contains an *rpo*A gene encoding the N-terminal His_6_-tagged α subunit of *E. coli* RNA polymerase (Tang *et al.* 1994).

### Generation of P_m_ mutants

Multiple mutations were introduced into P_m_ using polymerase chain reaction (PCR) primers containing degeneracy in specific targeted positions (Table 1) (Chiang and Howe 1993; Chiang *et al.* 1993). One set of primers was designed to be degenerate, using all four (N) bases in equal molar amounts (A_1_T_1_G_1_C_1_) during oligonucleotide synthesis. For synthesis of a second set of primers, the N mixture was supplemented with extra A+T (A_2_T_2_G_1_C_1_) or G+C (A_1_T_1_G_2_C_2_) to produce primers that were AT-rich or GC-rich. Incorporation rates for all the primers were approximately 0.5/nucleotide or 0.25/nucleotide at each position.

Two sequential PCR amplifications were performed. The first used wild-type (WT in Figures, Legends, and Tables) primer MLK7 and one degenerate primer (JM1-JM6 orJM40 or MEG5-MEG8) to create templates for the second PCR with wild-type primers MEG4 and MLK7, which contained *Eco*RI (MEG4) or *Bam*HI (MLK7) restriction sites near their 5′ ends. The resulting PCR products were digested with *Eco*RI and *Bam*HI and cloned into the *Eco*RI and *Bam*HI sites in the promoter-less vector pIA12. The ligation mixtures were transformed into MH13335, which contains pKM78, with P*_lac_*_UV5_-*mor*.

### Determination of MacConkey plate phenotypes

Transformants were plated on LB plates containing Ap and Cm, and single colonies were picked and stabbed in parallel onto LB plates and MacConkey lactose plates with 0.01, 0.1, and 1 mM IPTG. After incubation at 32° for 12 hr and/or 24 hr, the colony color was scored relative to that of MH15001 (WT P_m_, positive control) and MH13339 (no P_m_, negative control) on the same plate. Mutants that were red with less IPTG than that required for the wild-type were scored as Up, whereas mutants which required more IPTG than that needed for the wild-type to become red were scored as Down.

### *In vivo* transcription activation assays

Strains grown overnight in LB with Ap and Cm were inoculated into 3 mL of M9 minimal medium with 0.2% casamino acids at a 1:100 dilution. The cells were grown at 37° to an OD_600_ of 0.3−0.5. Then, IPTG was added to a final concentration of 2 mM to induce Mor production, and the cultures were grown at 37° for 1 hr. Uninduced control cultures were grown in parallel for all samples. Assays for β-galactosidase were carried out as described by Miller (1972) with minor modifications (Chiang and Howe 1993; Kumaraswami *et al.* 2004). The β-galactosidase activity was calculated in Miller units (Miller 1972) and normalized to the activity of a wild-type culture, which was assayed in parallel, and set to 1000 units. Enzymatic assays were performed in duplicate in each of three independent experiments, and the values were averaged to generate the activity reported.

### DNA binding assays

Radiolabeled probes used in gel retardation and footprinting assays were prepared by PCR as described previously (Ma and Howe 2004). They consisted of 198-bp probes containing P_m_ sequence from −98 to +10 and were generated by PCR using flanking vector primers IRI21 and IRI22, one of which was ^32^P-labeled. Plasmids containing wild-type (pMM1) or mutant P_m_ sequences were used as templates. The probes were purified using a Qiaquick spin purification kit and the DNA concentration estimated by comparison to a low-DNA-mass ladder.

Gel mobility shift assays for Mor binding were performed as described by Ma and Howe (2004) except that approximately half (~0.38 nM) the prior amount of DNA probe was used. Mor protein was diluted into binding buffer on ice immediately before use, then added to the binding reaction. The mixture was incubated for 15 min at 30° just before being subjected to native polyacrylamide gel electrophoresis. Gels were exposed to X-OMAT AR film (Kodak) without drying.

Gel mobility shift and DNase I footprinting assays using both Mor and N-terminal hexa-histidine-tagged α were performed as described previously (Ma and Howe 2004). The His-α protein was added after preincubation of the DNA probes with Mor for 10 min at 30°, and incubation was continued for an additional 10 min at 30° before polyacrylamide gel electrophoresis.

## Results

### Isolation of P_m_ mutants

**Figure 3:**
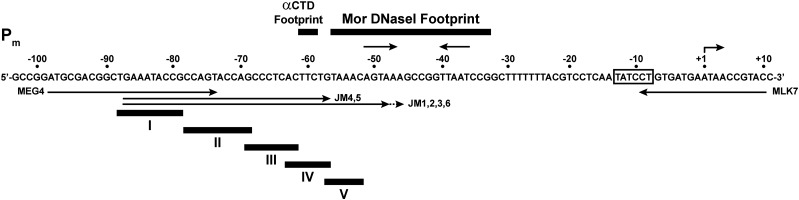
Regional mutagenesis of P_m_ sequence upstream of the Mor binding site. The P_m_ promoter sequence is shown from −103 to +11 with respect to the transcription initiation site as +1. Dots above the sequence indicate 10-bp intervals that are numbered by their position in the promoter with respect to +1. The −10 sequence is in a box and +1 is indicated by the bent arrow. The bold lines above the sequence indicate the positions of the DNase I footprints for Mor and for the αCTD. The short inverted arrows above the sequence identify the locations of the 5-bp inverted repeats of the Mor binding site. The long thin arrows below the sequence show the P_m_ bases present in the primers used for mutagenesis. The bold lines below the sequence show the regions I-V that were mutagenized.

The goal of this study was to determine whether P_m_ contains an UP element. Our approach was to isolate and characterize P_m_ mutants with base changes in the region upstream of the Mor binding site. The mutagenesis strategy was to divide the P_m_ sequence upstream of the Mor binding site into multiple regions based on their GC-content, helix face, and proximity to the Mor binding site (Figure 3). Mutagenesis of each region was performed separately using degenerate oligonucleotide primers (Table 1). For regions I (−88 to −79) and II (−78 to −69), the primers were designed to change the targeted region to be more A/T- or G/C-rich. For regions III (−69 to −62), IV (−63 to −57), and V (−57 to −52), a random mixture of all four bases was used at targeted positions. PCRs were performed first with the degenerate oligonucleotide primers and an opposing wild-type primer (MLK7) to produce a template for a second PCR with overlapping wild-type primers (MEG4 and MLK7). This second PCR completed the promoter sequence and provided the *Eco*RI and *Bam*HI restriction sites for cloning. The PCR products were cloned between the *Eco*RI and *Bam*HI sites in the promoter-less *lacZ* fusion vector pIA12 (Figure 2) just upstream of the *lacZ* gene. The ligation mix was transformed into strain MH13335 containing the activator plasmid pKM78, which expresses Mor when induced by IPTG (Figure 2). The transcription activities of the mutant promoters were examined by color development of each transformant on MacConkey lactose plates with different concentrations of IPTG, and scored as Up, Down, or wild-type relative to the color developed by the control strain (MH 15001) containing a plasmid with wild-type P_m_ on the same plate.

Mutagenesis of P_m_ sequences for regions I through IV resulted in transformant populations with a mixture of wild-type, Up, or Down phenotypes; however, all of the candidate Up and Down mutants had an aberrant or missing P_m_ fragment and were not characterized further. We then sequenced the promoters in transformants that exhibited wild-type phenotypes to demonstrate that the mutagenesis was effective and to identify bases necessary and unnecessary for promoter activity. The results showed that these clones contained an average of at least 0.68 mutations per targeted base, and thus an average of 6−7 base changes per mutant plasmid (Table 2). Having mutated each base multiple times with no detectable effect, we concluded that none of the bases in these targeted regions (I−IV) were required for P_m_ activity.

**Table 2:** Phenotypes of transformants containing mutations in P_m_ upstream regions

Region*^a^*	I		II		III		IV	
	–88 to –79		–78 to –69		–69 to –62		–63 to –57	
Positions changed	−88 to –79 to GC-rich	−88 to –79 to AT-rich	−78 to –69 to GC-rich	−78 to –69 to AT-rich	−69 to –62*^b^*	−65 & –63	−62, –59 & –57	−63, –61, –60 & –58
Total scored	24	34	33	42	250	50	69	43
Down (%)*^c^*	0	0	0	0	0	0	0	0
Up (%)*^c^*	0	0	0	0	0	0	0	0
WT (%)*^c^*	100	100	100	100	100	100	100	100
Number sequenced	12	10	11	9	14	34	22	25
Mutations per bp	0.77	0.68	0.82	0.77	0.69	0.84	0.68	0.75

a Transformants for regions I and II were made and assayed by Meghan Mitchell.

b Phenotypes for this group of transformants were only examined on MacConkey lactose plates with 0.1 mM IPTG.

c Percentages reflect the phenotypes for mutants with an intact, but mutant, promoter.

For region V, two different mutagenesis strategies were used. In one, all the bases −57 through −52 were mutated. In the other, only positions−57 and −52 were mutated, because they were already known to have some effect (Artsimovitch and Howe 1996). The majority of these transformants exhibited a range of reduced red color on the MacConkey plates and had one or more base substitutions in region V.

Quantitative analysis of promoter activity was determined for representative mutants in all five regions by performing *in vivo* liquid β-galactosidase assays. Mutants with promoter activity in the range of 80–120% of the wild-type were grouped with the wild-type, because that level of variability could be found with independent assays of a single culture (Chiang and Howe 1993). The results were completely consistent with those from the MacConkey plate assays. Mutants with mutations in regions I through IV had wild-type activity. Mutants with mutations in region V had a range of β-galactosidase activities, most exhibiting only 10–50% of the wild-type activity.

Sequence and promoter activity of mutants with mutations in region V (−57 to −52) are shown in Table 3; note the grouping of bases at −52 and −53. These results demonstrated the following: (1) Substitutions of C at position −52 by A or T resulted in an approximately 50% reduction in P_m_ activity, whereas substitution of C by G did not significantly affect promoter activity. (2) In mutants with C or G at −52, all three possible substitutions of the base at position −57 had little or no effect on P_m_ activity. (3) Because mutants with wild-type bases at both −57 and −52 had greatly reduced activity, we conclude that one or more bases at positions −56 to −53 are important for promoter activity.

**Table 3:** Promoter activity*^a^* of P_m_ upstream mutants with mutations in region V

Strain	–57	–56	–55	–54	–53	–52	β-gal Activity*^a^*	SD^b^
A								
WT*^c^*	G	T	A	A	A	C	1000	
JM1–1	C					A	536	51
JM1–7	A					A	589	69
JM1–14						A	414	57
JM1–2	A					T	550	116
JM1–3						T	407	89
JM1–5	T					T	642	73
JM1–6						T	434	65
JM1–11	C					T	696	50
JM1–16	T					G	856	63
JM1–18						G	982	69
JM1–20	C					G	1003	87
JM1–21	A					G	924	94
JM1–25	C						879	64
B								
WT	G	T	A	A	A	C	1000	
JM6–103		G					1015	66
JM6–12	A	G	T	T			416	82
JM6–25	C	A	G	G			473	54
JM6–105	A	G	G	T	C		64	22
JM6–104	T	A	G	T	C		67	16
JM6–59		A	C		C		247	78
JM6–4	A		G		G		538	77
JM6–23	T		C		G		421	80
JM6–33		G		G	G		338	74
JM6–108		G	G	T	G		245	96
JM6–24	T	C	G	T	G		494	62
JM6–73	A	A	G	C	T		273	66
JM6–95	C	A	G	C	T		287	53
JM6–34		A	T	C	T		390	50
JM6–86	T	C		C	T		456	117
JM6–70	T	C	T	G	T		358	119
JM6–1	C	G	G	G	T	G	306	96
JM6–26		A	T	G	T	G	350	81
JM6–10		G	G		T	G	415	42
JM6–17	T		G	T	T	G	451	94
JM6–5	T		T	T		G	402	31
JM6–87		G		G		G	731	32
JM6–22	C		G	G		G	362	84
JM6–14				G	G	G	430	81
JM6–35	A	C	C	G	G	G	442	43
JM6–88				T	G	G	495	168
C								
WT	G	T	A	A	A	C	1000	
JM6–11	T	A		T	C	G	367	141
JM6–36		C	T	T		T	398	61
JM6–80	A	A	G	C		T	191	18
JM6–16		A		T	G	T	414	5
JM6–21		G		T	G	T	302	82
JM6–2	A			G	G	T	349	129
JM6–7		G			T	T	518	80
JM6–15		G	T		T	T	542	114
JM6–90		A	G	T	T	T	593	107
JM6–106		G	G	T	C	T	39	11
JM6–8	T	G	G	T	G	A	356	118
JM6–6	C	G	T	T	G	A	530	72
JM6–93		G	T	T		A	837	143

a Promoter activity was assayed by determining β-galactosidase (β-gal) levels in liquid cultures after induction of Mor synthesis in cells containing the P_m_-*lacZ* fusions. Assays were performed in duplicate in at least three experiments for each mutant in parallel with the wild-type (WT) strain MH15001. In each experiment the activities for the mutants were normalized relative to the closest WT row above them, which was set to 1000. The activity shown here is the average of the results from those three experiments.

b SD represents standard deviation.

c The results for strains from different experiments A, B, and C are separated by blank rows.

### Analysis of P_m_ mutants by gel mobility shift and DNase I footprinting assays

**Figure 4:**
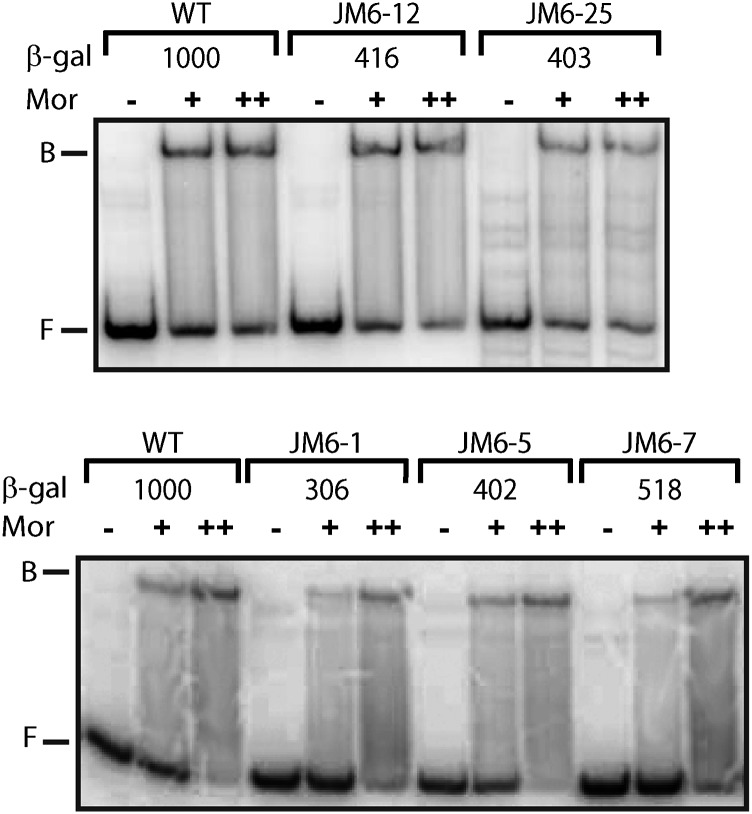
Representative results for gel retardation analysis of Mor binding to P_m_ DNA fragments with upstream mutations. Radiolabeled WT or mutant P_m_ probes containing P_m_ sequence −98 to +10 (at ~0.4 nM) were incubated with 0 (−), 68 nM (+), or 340 nM (++) purified Mor protein in 20 μL of binding buffer (Ma and Howe 2004) for 15 min at 30°. Binding mixtures were loaded onto 8% native acrylamide gels (29:1) in 0.5x Tris/Borate/EDTA buffer and subjected to electrophoresis for 3 hr at 4° and 15V/cm. The positions of free probe and bound complexes are indicated by F and B, respectively.

Because mutations close to the Mor binding site (−51 to −36) could affect P_m_ activity by altering Mor binding, gel mobility shift assays were performed to examine Mor binding to mutant P_m_ DNA fragments. All of the mutants had substantial Mor binding as shown in the representative gels presented in Figure 4.

Previous DNase I footprinting analysis showed that Mor alone protected P_m_ positions from −56 to −33 from digestion by DNase I (Artsimovitch *et al.* 1996; Kahmeyer-Gabbe and Howe 1996; Ma and Howe 2004). Addition of RNA polymerase led to an extension of the footprint to +14 and produced a new footprint from −61 to −59 upstream of the Mor-binding site (Artsimovitch *et al.* 1996; Ma and Howe 2004). Addition of α or the αCTD instead of RNA polymerase resulted in the same −61 to −59 footprint, demonstrating that this footprint is caused by αCTD binding (Ma and Howe 2004). To examine whether mutations in region V affected αCTD binding, we performed DNase I footprinting of the mutant promoters with Mor and RNA polymerase. Open complexes formed by Mor plus RNAP have a different footprint from those produced using α or the αCTD; therefore, these assays were done at 15° to prevent open complex formation. In these experiments saturating or almost saturating concentrations of Mor protein (860 nM) were used, and each DNA probe assayed contained a Mor-only lane for comparison.

**Figure 5:**
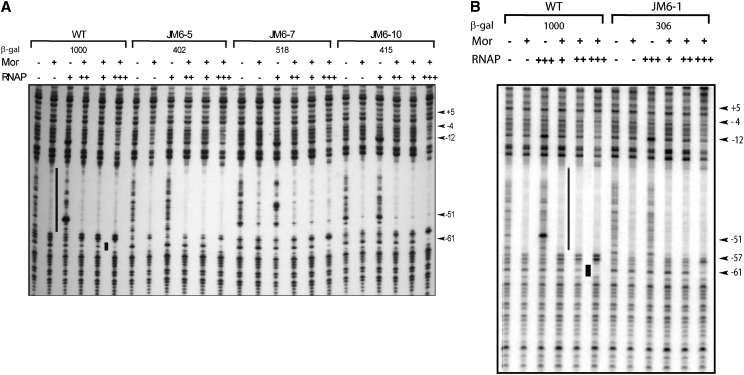
DNase I footprinting of P_m_ at 15°. Linear 5′ end-labeled probes containing P_m_ sequence from −98 to +10 flanked by pIA12 vector sequences were made by PCR using labeled vector primer IRI 21, unlabeled vector primer IRI 22, and plasmids containing WT or mutant P_m_ as templates. Probe (~0.8 nM) was incubated with 860 nM Mor (+) alone, 50 nM RNAP (+++) alone, or with Mor at 860 nM plus RNAP at 10 nM (+), 20 nM (++), or 50 nM (+++) at 15° before DNase I digestion. The region protected by Mor is indicated with a vertical black line, and the upstream footprint is shown by a black rectangle. The numbers on the right correspond to positions in the P_m_ sequence relative to the start site +1. The names for the P_m_ mutant probes are indicated at the top of each panel; (A) probes containing WT P_m_ and P_m_ mutants JM6-5, JM6-7, and JM6-10 and (B) probes containing WT and mutant JM6-1.

The footprinting patterns of Mor alone with wild-type or mutant DNA probes (Figure 5) were the same as observed previously (Artsimovitch *et al.* 1996; Ma and Howe 2004). Reactions with RNA polymerase alone produced little or no protection but did exhibit hypersensitive bands at positions −51 and −12, demonstrating the presence of unstable or weak interactions between RNAP and P_m_ DNA, as observed previously by Mo and Howe (2014). The upstream mutations had no effect on the −12 hypersensitivity, presumably because it reflects interaction of RNAP with the −10 hexamer. In contrast, none of the mutant probes showed the same high degree of hypersensitivity at band −51. There are two possible explanations: first, the interactions between RNAP and promoter DNA was affected by the mutations, leading to differences in the footprint patterns or second, the local structure of the DNA was changed by the mutations, affecting the accessibility of DNase I to the DNA, thereby altering the footprint pattern. When both Mor and RNA polymerase were present, the −61 to −59 region of P_m_ was protected. With increasing concentrations of RNA polymerase, this protection became more complete. At the same time, a very weak protection was observed extending downstream to −4. Protection to −4 is characteristic of a closed complex in which RNAP binding is unstable.

### P_m_ mutants containing mutations at selected sites

The aforementioned DNase I footprinting assays using P_m_ region V mutants affected the degree of protection from −61 to −59 but did not indicate which bases were most important. To address this question, we made several additional mutants containing specific targeted mutations. Because mutations of adenines at positions −41, −42, and −43 in the *rrnB* P1 UP element proximal subsite, corresponding to adenines at positions −53, −54, and -55 at P_m_, led to dramatic decreases in promoter activity (Estrem *et al.* 1999; Ross *et al.* 2001), and to avoid mutating positions that might lead to decreased Mor binding (−52, −53), we made five new plasmids containing mutations upstream of −53.

Plasmid pJM7 contains mutations−57GA and −56TA, forming an A5 tract extending from positions −57 to −53, matching the preferred binding sequence of the αCTD. Plasmids pJM8, pJM9, and pJM10 all contain mutations at positions −55 and −54, where both As were changed to G or C, mutations expected to disrupt αCTD-DNA interactions. In addition to mutations at −55 and −54, plasmid pJM11 contains mutations of Cs to As at positions located one helical turn upstream from the present αCTD-binding site and expected to create a good distal αCTD-binding site.

### Activity assay *in vivo*

Plasmids pJM7-pJM11 were transformed into strain MH13335, and liquid β-galactosidase assays were performed to quantitate the activities of the mutant promoters. A summary of the results is shown in Table 4, which also contains data for several of the earlier plasmids with multiple mutations for comparison. Mutation of bases at positions −56 and −57 in pJM7 to form an A5 tract did not significantly increase or decrease promoter activity, suggesting that the wild-type P_m_ sequence is already strong. The three mutants (pJM8, 9, 10) containing G or C mutations at both positions −55 and −54 in the native A3 tract showed substantial reductions in P_m_ activity to 22%, 46%, and 19% of wild-type, respectively. The β-galactosidase activities expressed from previous mutants JM6-73, JM6-80, and JM6-95 are similar to that from pJM8, suggesting that the two bases −55AG and −54AC are the most important in reducing P_m_ activity. The mutants JM6-1, JM6-22, and JM6-25 share mutations −55AG and −54AG with pJM9 and had similar promoter activities, with only modest effects from their additional mutations. In contrast, the mutant pJM7 with substitutions −57GA and −56TA had an activity similar to WT, and the same two substitutions in mutants JM6-73 and JM6-80 were not able to compensate for the deleterious effects of their additional mutations. The mutant pJM11 containing mutations that moved the αCTD-binding site one helical turn upstream had the largest defect, making only 15% of wild-type activity, implying that the location of the αCTD-binding site in P_m_ is important, possibly because the αCTD requires direct interaction with Mor.

**Table 4:** Results and comparison of *in vivo* promoter activity*^a^* assays

Plasmid Name	–68 to –62	–57	–56	–55	–54	–53	–52	β-gal Activity*^a^*	SD*^b^*
WT	CCCTCAC	G	T	A	A	A	C	1000	
**pJM7***^c^*		**A**	**A**					**884**	**105**
**pJM8**				**G**	**C**			**224**	**53**
JM6–80*^d^*		A	A	G	C		T	191	18
JM6–73		A	A	G	C	T		273	66
JM6–95		C	A	G	C	T		287	53
**pJM9**				**G**	**G**			**459**	**56**
JM6–1		C	G	G	G	T	G	306	96
JM6–22		C		G	G		G	362	54
JM6–25		C	A	G	G			473	54
**pJM10**				**C**	**C**			**188**	**18**
**pJM11**	**AAATAAA**			**C**	**C**			**146**	**33**

a Liquid β-galactosidase (β-gal) assays were performed as described in Table 3.

b SD represents standard deviation.

c Sequences and results of β-galactosidase assays for the new double mutants pJM7-pJM11 are shown in bold type.

d Sequences and results of β-galactosidase assays for relevant mutants from the JM6 series are copied here from Table 3 to facilitate comparison of their activities.

### DNase I footprinting and gel mobility shift assays with promoter DNA from plasmids pJM7 to pJM11

**Figure 6:**
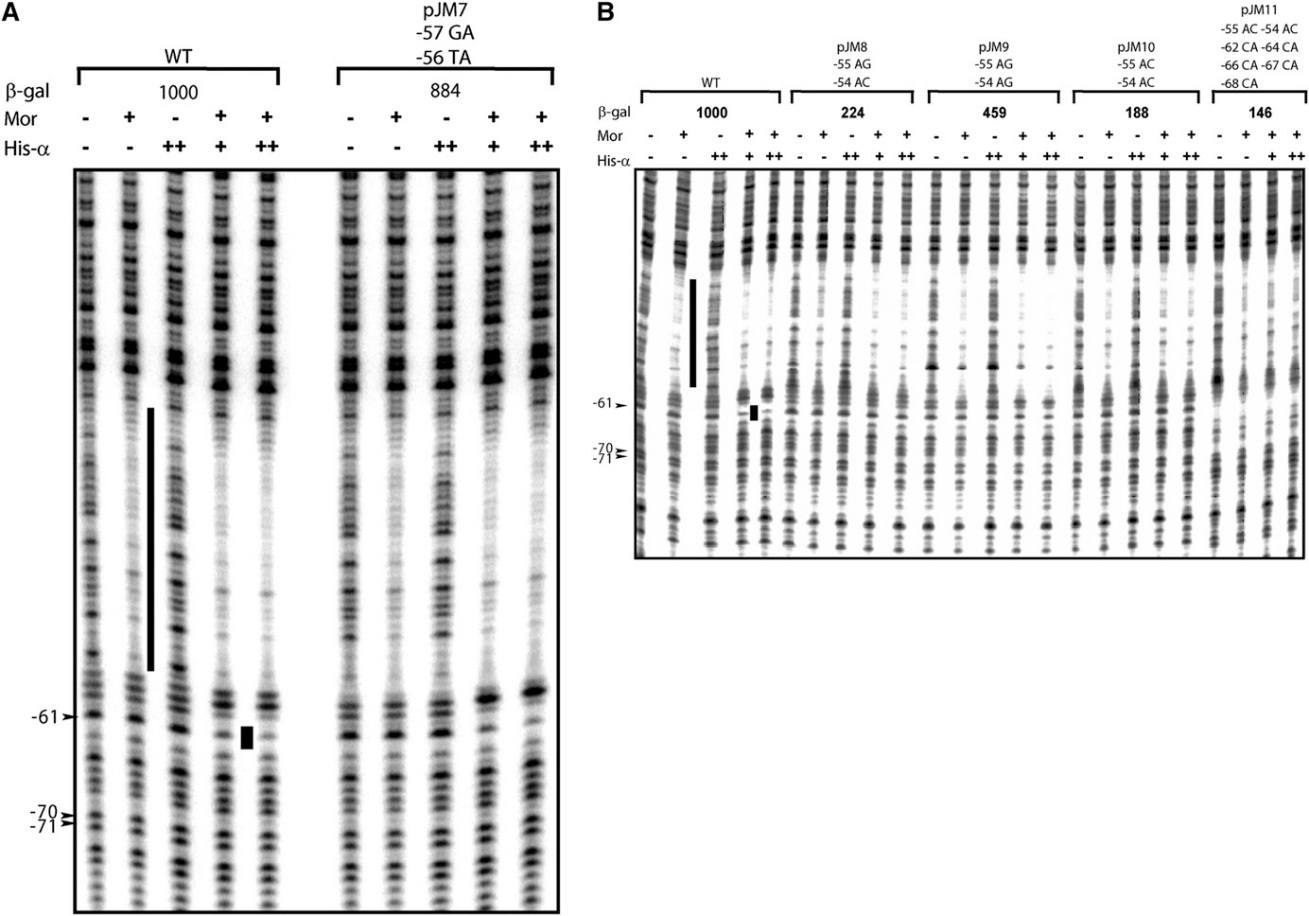
DNase I footprinting analysis of WT and mutant P_m_ DNA using purified His-α. Linear end-labeled probe containing P_m_ sequence from −98 to +10 flanked by pIA12 vector sequences was made by PCR using labeled primer IRI 21, unlabeled primer IRI 22, and plasmid pMM1 as template. Probe (~0.8 nM) was incubated with either 850 nM Mor or 2.6 μM (++) His-α for 10 min at 30°. For the synergistic binding assays, Mor was incubated with probe for 5 min before the addition of 1.3 μM (+) or 2.6 μM (++) His-α. The mixture was incubated for another 10 min at 30° before DNase I digestion. The region protected by Mor is indicated with a long vertical black line, and the upstream footprint is indicated by a black rectangle. The numbers on the left correspond to positions in the P_m_ sequence relative to the start site +1. Plasmid names and altered sequence are labeled at the top of each panel as follows: the number indicates the position of the mutagenized base, the first letter indicates the WT base and the second letter indicates the mutant base. (A) Probes were made from WT or mutant plasmid pJM7. (B) Probes were made from WT or mutant plasmids pJM8-pJM11.

The mutations in pJM7-pJM11 were designed not to affect Mor binding. As expected, gel mobility shift assays with these mutants showed wild-type levels of Mor binding (data not shown), allowing DNase I footprinting assays to be carried out to examine His-α binding without different levels of Mor binding as a confounding variable. The DNase I footprinting pattern of the pJM7 probe, containing mutations −57GA and −56TA and making almost wild-type levels of β-galactosidase was very similar to that of the wild-type probe (Figure 6A); a slight footprint at position −59 and a more pronounced footprint at −61 were generated when Mor and His-α were both added. The pJM8 and pJM10 probes containing −55AG and −54AC mutations (pJM8) or −55AC and −54AC mutations (pJM10) showed little or no footprint at −59 and −61, indicating a substantial reduction in His-α binding (Figure 6B). These results are consistent with the substantially lower β-galactosidase activities of these mutant plasmids. The probe pJM9 containing −55AG and −54AG mutations showed a His-α binding ability intermediate between that of the wild-type probe and that of pJM8 and JM10, consistent with its *in vivo* transactivation assay which was slightly less than half of the wild-type β-galactosidase activity. When pJM11 was assayed, it had no −61 band, presumably due to the multiple mutations it carries, and showed no changes in band pattern beyond those caused by Mor binding, providing no evidence for or against His-α interaction with the DNA.

**Figure 7:**
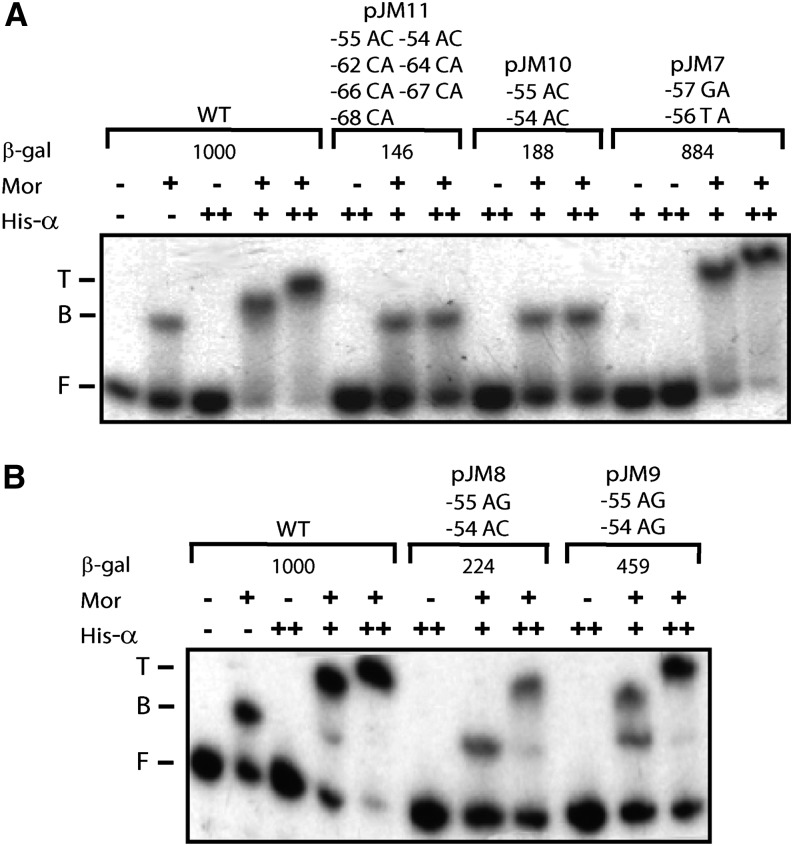
Gel retardation analysis of His-α and Mor binding to WT and mutant P_m_ DNA. Radiolabeled DNA probes (~0.4 nM) containing P_m_ sequence −98 to +10 were incubated with 136 nM Mor or 2.7 μM (++) His-α for 10 min at 30°. When both proteins were used, Mor was incubated with the probe for 10 min, His-α was added to a final concentration of 1.3 μM (+) or 2.7 μM (++), and a second incubation for 10 min at 30° was performed. Binding mixtures were then loaded onto 4% acrylamide native gels containing 5% glycerol and 0.5x Tris/Borate/EDTA (TBE) buffer and subjected to electrophoresis in 0.5x TBE buffer containing 2% glycerol at 3°. The name of each mutant plasmid template and the mutations in each is given above each bracket. The positions of free probe, Mor-DNA binary complex, and Mor-His-α-DNA ternary complexes are indicated with F, B, and T, respectively. Note the unusual behavior of the ternary complex: the greater the His-α concentration, the slower the mobility of the complex. This behavior is characteristic of α whether or not it is tagged. In fact, when multiple concentrations are used, each increased concentration yields a slower band (Savery *et al.* 1998**).** An explanation for this behavior has not been determined.

To examine the effects of the pJM7-pJM11 mutations on synergistic binding of His-α and Mor to P_m_, we carried out gel mobility shift assays with purified His-α and Mor (Figure 7). When wild-type probes were used, no shift was observed for His-α alone even at a high concentration of His-α (2.7 μM), and only one-third to one-half of the probe was shifted with Mor alone. In contrast, when both proteins were added, the wild-type DNA probes were almost completely found in a supershifted species corresponding to a Mor-His-α-DNA ternary complex, even at a low concentration of His-α (1.3 μM). When mutant probes were used, different shifting behaviors were observed. Probe containing mutations at positions −57 and −56 (pJM7) gave a shift similar to the wild-type probe, consistent with its wild-type β-galactosidase activity. When probes containing −55AC and −54AC mutations (pJM10 and pJM11) were used, only a binary complex of Mor and probe was observed, even at a greater concentration of His-α (2.7 μM), suggesting that these mutations disrupted interactions between the αCTD and DNA, which is consistent with their very low β-galactosidase activities. The probe containing −55AG and −54AC mutations (pJM8) showed reduced synergistic binding, relative to the wild-type probe. At the lower concentration of His-α (1.3 μM), there was no supershifted complex, and when more His-α (2.7 μM) was used, only a small fraction of the probe was seen in a supershifted complex. The probe containing −55AG and −54AG mutations (pJM9) showed a shifting ability intermediate between the wild-type probe and the pJM10 and pJM11 probes containing A to C substitutions at these positions. For pJM9, at 1.3 μM His-α, both binary and ternary complexes were observed and when a higher concentration of His-α (2.7 μM) was used, more than half of the probe was seen in the supershifted species.

## Discussion

This study demonstrated that the DNA sequence in the region immediately upstream of the Mor-binding site in P_m_ had a substantial influence on promoter activity and α DNA binding, both characteristics of an UP element. When mutations designed to destroy an UP element were used, *i.e.*, substitutions of −55A and −54A by cytosines (pJM10, Figure 7), there was a more than 80% decrease in P_m_ activity and extremely decreased binding of α.

Interactions between α (*i.e.*, the αCTD) and DNA can be sequence-dependent or sequence-independent (Ross *et al.* 1993; Busby and Ebright 1999; Gourse *et al.* 2000). At promoters containing strong UP elements, for example, the *rrnB* P1 promoter, α binds to DNA in the absence of activators, and mutation of these high-affinity DNA sequences reduces αCTD binding. At other promoters, α binds to DNA only in the presence of activators. At these promoters, the interaction between α and DNA is only partially sequence-dependent, and the binding site for α is usually located adjacent to the activators (Flatow *et al.* 1996; Czarniecki *et al.* 1997). At P_m_, α binds just upstream of the Mor-binding site, as it does at the consensus promoter CC (−41.5), the best-characterized Class II CAP-dependent promoter (Savery *et al.* 1998). When this α-DNA interaction was disrupted by mutations −55AC and −54AC, α binding to P_m_ was undetectable even in the presence of Mor (pJM10 in Figure 7). When the α-DNA interaction was only partially disrupted, α still bound to P_m_, but with lower affinity (pJM8 in Figure 7).

At the *rrnB* P1 promoter and some CAP-dependent promoters, α binding sites can be relocated upstream by one or two helical turns without dramatically affecting promoter activity. Nevertheless, the binding site closest to the core promoter is preferred (Lloyd *et al.* 1998; Estrem *et al.* 1999; Newlands *et al.* 1991). When we tried to destroy the current α binding site in P_m_ and produce a new favorable α binding site one helical turn upstream (pJM11), we observed a dramatic decrease in both P_m_ activity and α-DNA binding. It is possible that binding of α further upstream caused a loss of favorable interactions between Mor and α, or that binding of α further upstream put constraint on the linker between the αCTD and the αNTD, making binding less energetically favorable.

Mutations in the α binding site most likely affected P_m_ activity by reducing stable binding of α to P_m_, as suggested by DNase I footprinting and gel shift assays. Less Mor-DNA-His-α ternary complex was formed when P_m_ mutations designed to reduce α-DNA binding were used. This demonstrated that interactions between Mor and α were not strong enough to overcome the lack of α-DNA interactions. Thus, interactions between Mor and α are mutually stabilized by the interactions between α and the promoter DNA. Full activation of P_m_ most likely requires Mor-α interactions as well as Mor-DNA and α-DNA interactions.

These results led us to conclude that the bases immediately upstream of the Mor binding site constitute a promoter-proximal subsite of an UP element, but these sequences lack a promoter-distal subsite.
